# Supplementary figures and images for: Early embryogenesis and organogenesis in the annelid Owenia fusiformis
Source: EvoDevo. 2021 May 10;12:5. doi: 10.1186/s13227-021-00176-z (PMC8111721; doi:10.1186/s13227-021-00176-z)

**a**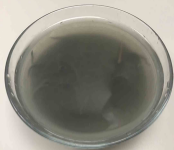**b**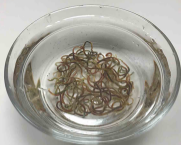**c**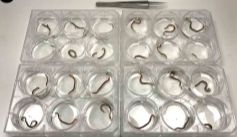**d**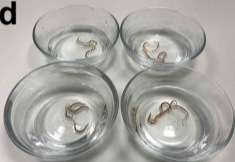**e**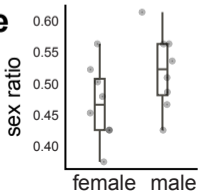**f**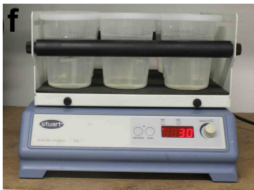

Supplement: Supplementary file 2 — Additional file 2: Figure S1. Culture and artificial fertilization. Adults are (a) relaxed with MgCl2 and (b) stripped from their tubes. (c) Adults are sorted and (d) separated by sex. (e) The sex ratio of adults used in this study was kept very similar. (f) After 27 hpf, the larvae were transferred to 600 ml plastic beakers and grown at 15 °C. [file 13227_2021_176_MOESM2_ESM.pdf]

a

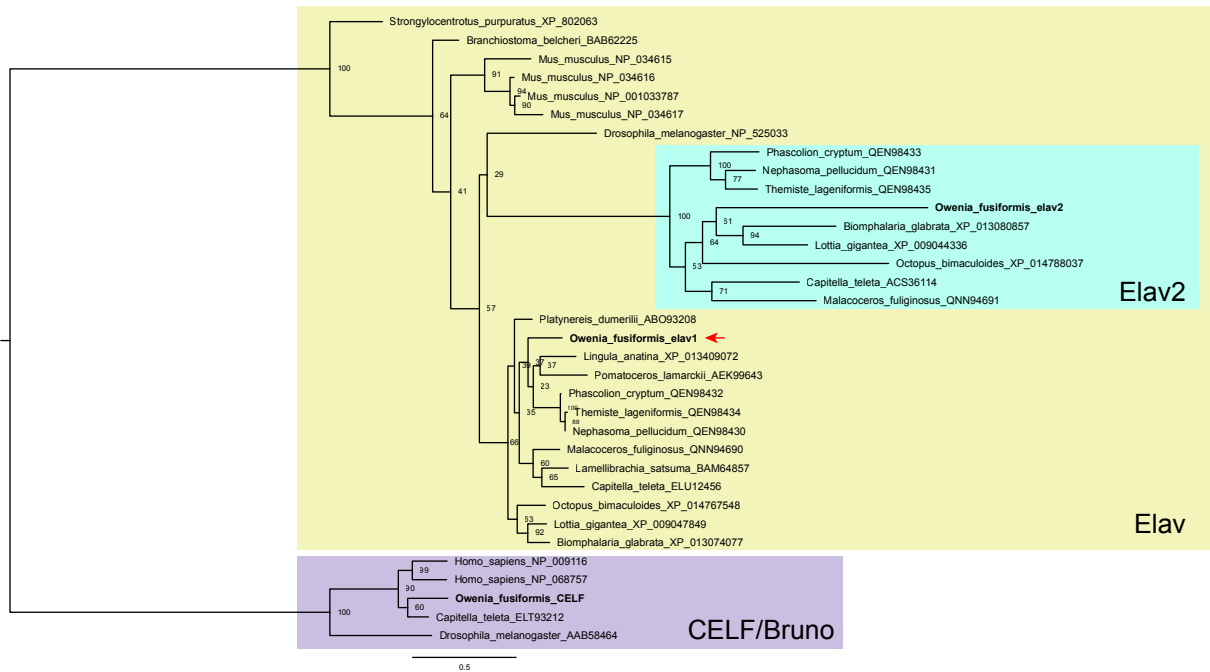

b

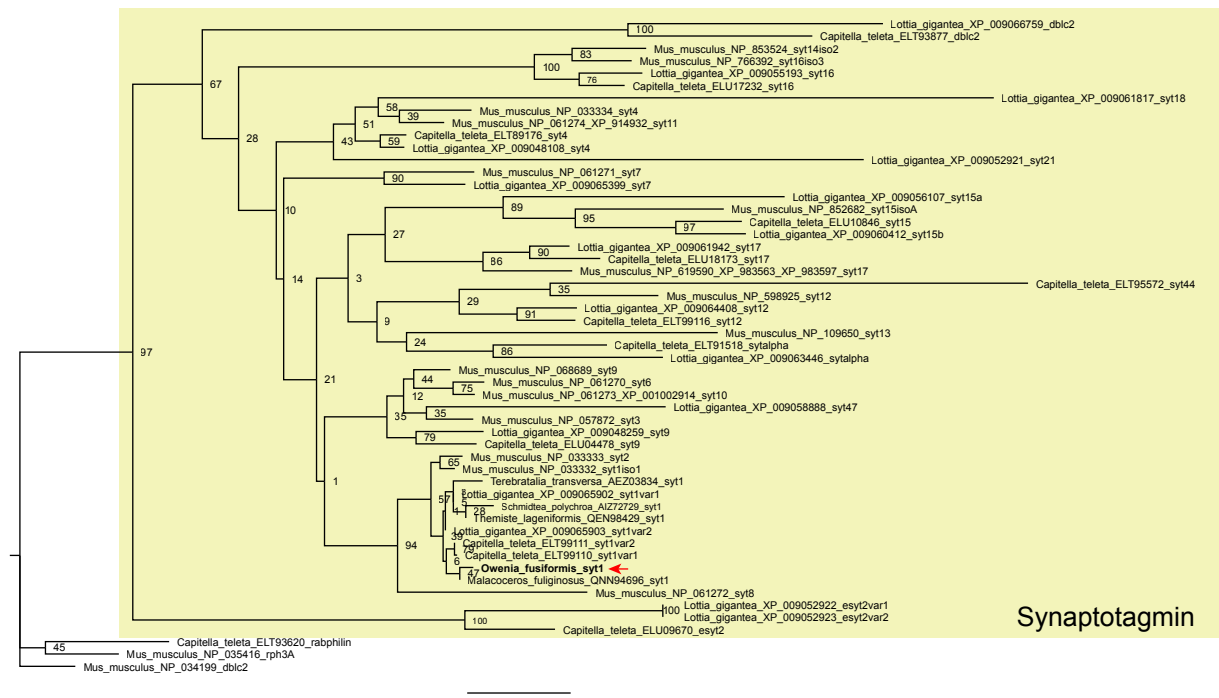

Supplement: Supplementary file 3 — Additional file 3: Figure S2. Phylogenetic relationships of O. fusiformis Elav1 and Synaptotagmin1 proteins. (a) RaxML phylogenetic tree of Elav and (b) Synaptotagmin1 of O. fusiformis. (a) Similar to other spiralians, there are two Elav proteins in O. fusiformis. (b) O. fusiformis Synaptotagmin1 clusters with the Synaptotagmin1 clade from other spiralians. Refer to the methods for the specifics of the phylogenetic analyses and Additional file 4: Table S2 for the accession numbers used. [file 13227_2021_176_MOESM3_ESM.pdf]
